# The C‐terminal truncated splicing variant of NK1R negatively modulates substance P‐stimulated NK1R signaling

**DOI:** 10.1002/2211-5463.70334

**Published:** 2026-09-21

**Authors:** Lan Phuong Nguyen, Duc Trung Nguyen, Minyoung Cho, Jihun Kim, Soyeon In, Thai Uy Nguyen, Sunghoon Hurh, Beom Jin Park, Jong‐Ik Hwang

**Affiliations:** ^1^ Department of Biomedical Sciences, College of Medicine Korea University Seoul Republic of Korea; ^2^ Department of Radiology, College of Medicine Anam Hospital Korea University Seoul Republic of Korea

**Keywords:** migration, neurokinin 1 receptor, receptor dimerization, splicing variants, substance P

## Abstract

Mammalian neurokinin 1 receptor (NK1R) exists as full‐length (NK1L) and truncated (NK1S) splice variants. However, the functional role of NK1S remains controversial; therefore, we investigated their functional interplay. Using NanoBiT and co‐immunoprecipitation, we demonstrate that NK1L and NK1S form heterodimeric complexes. Through this interaction, NK1S negatively modulates NK1L‐mediated G protein signaling, specifically impairing Gαq coupling and Ca^2+^ mobilization. Conversely, NK1S enhances β‐arrestin1 recruitment to NK1L, altering receptor trafficking. In A549 cells, NK1S suppressed NK1L‐mediated cell migration despite sustaining ERK phosphorylation. These findings provide mechanistic evidence that NK1S can regulate NK1L through dimerization and may shift signaling bias from G proteins toward β‐arrestin‐linked pathways to influence cellular outcomes.

## Graphical abstract

The neurokinin 1 receptor exists as full‐length (NK1L) and C‐terminally truncated (NK1S) splice variants. We show that NK1S heterodimerizes with NK1L, impairing Gαq coupling and Ca^2+^ mobilization while enhancing β‐arrestin1 recruitment. NK1S suppresses substance P‐driven gene expression and cell migration, revealing NK1S as an endogenous biased modulator that shifts NK1L signaling from G proteins toward β‐arrestin.

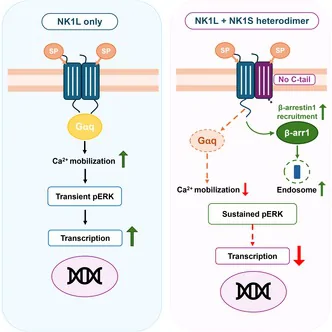

**Abbreviations**
CMV: cytomegalovirus
CRE: cyclic AMP response element
DAG: diacylglycerol
ERK: extracellular signal‐regulated kinase
GPCR: G protein‐coupled receptor
HA: hemagglutinin
IP3: inositol trisphosphate
LgBiT: large fragment of NanoLuc® luciferase
Luc: luciferase
NanoBiT: NanoLuc® Binary technology
NF‐κB: nuclear factor‐kappa B
NK1L: long form of neurokinin 1 receptor
NK1R: neurokinin 1 receptor
NK1S: short form of neurokinin 1 receptor
PIP2: phosphatidylinositol 4,5‐bisphosphate
PLC: phospholipase C
SmBiT: small fragment of NanoLuc® luciferase
SP: substance P
SRE: serum response element
UbiC: ubiquitin C

Substance P (SP) is an 11‐amino acid oligopeptide (Arg‐Pro‐Lys‐Pro‐Gln‐Gln‐Phe‐Phe‐Gly‐Leu‐Met‐NH_2_) that belongs to the highly conserved tachykinin family. Originally identified for its role in neurotransmission, SP has since been recognized as a multifunctional neuropeptide involved in a wide range of physiological processes [1]. In mammals, SP is widely distributed throughout the body. It is expressed not only in neuronal cells, but also in other cell types such as fibroblasts, immune cells, and endothelial cells. SP plays critical roles in several physiological and pathological processes, including nociception, mood regulation, hematopoiesis, wound healing, inflammation, and cancer [2, 3, 4].

SP primarily exerts its biological functions by binding to the neurokinin 1 receptor (NK1R), a member of the class A G protein‐coupled receptor (GPCR) family. NK1R is broadly expressed in the nervous system, immune cells, and peripheral tissues, where SP/NK1R signaling mediates diverse physiological responses including pain transmission, neurogenic inflammation, and immune cell activation [5]. In pathological contexts, dysregulated SP/NK1R signaling has been implicated in chronic pain, depression, emesis, and cancer progression. Upon SP binding, NK1R couples predominantly to Gαq, triggering intracellular Ca^2+^ mobilization and activation of downstream effectors such as the MAPK/ERK pathway, NF‐κB, and SRE‐mediated gene expression, which are collectively linked to cell survival, proliferation, and migration [6]. NK1R can also engage Gαs to elevate cAMP levels and activate CRE‐binding protein, providing additional transcriptional outputs [7]. Sustained SP stimulation initiates receptor desensitization through GRK‐mediated phosphorylation of the receptor C‐terminal tail, followed by β‐arrestin recruitment, which uncouples the receptor from G proteins and facilitates receptor internalization into endosomes. This desensitization‐internalization cycle is a critical regulatory mechanism that determines the duration and magnitude of NK1R‐mediated signaling and is directly relevant to the functional divergence between NK1R splice variants examined in the present study.

The presence of two splicing variants of NK1R in mammals may complicate the deduction of its functional roles. These variants differ primarily in their C‐terminal regions. The full‐length form (NK1L) consists of 407 amino acids, while the truncated form (NK1S) consists of 311 amino acids that are identical to those of the full‐length form and lacks the C‐terminal region [8]. The C‐terminal intracellular region of GPCRs plays a crucial role in mediating interactions with intracellular signaling molecules, including G proteins, β‐arrestin, and GPCR kinases (GRKs) [9]. The absence of the C‐terminal region in NK1S may significantly affect the receptor's signaling capabilities and intracellular trafficking. In addition, splicing may influence tissue distribution of NK1R. NK1L is predominantly expressed throughout the central nervous system, while NK1S is detected in more restricted regions, including certain brain areas such as the cerebral cortex and is the dominant isoform expressed in peripheral immune cell types including monocytes and macrophages [10, 11, 12], with tissue‐specific NK1L‐NK1S ratios implying context‐dependent coregulation of SP signaling. These expression patterns suggest that NK1L and NK1S may have distinct physiological and pathological roles in specific regions. Upregulation of SP and NK1L is frequently observed and associated with several cancer types. Numerous studies have reported that NK1L facilitates tumor proliferation, angiogenesis, and metastasis, highlighting its critical role in the progression of several cancer types, including lung cancer, gallbladder cancer, leukemia, and colorectal cancer [4, 13, 14, 15, 16]. However, the biological roles of NK1S remain controversial. Lai et al. demonstrated the functional difference between NK1L and NK1S in terms of the activation of some signaling pathways, suggesting that NK1S may be a weaker mediator than NK1L [17]. Although this C‐terminal truncated form is thought to be deficient in mediating signaling, some studies have suggested that NK1S may also play an important role in cancer progression, particularly in promoting metastatic behavior [18, 19, 20]. Also, both isoforms are co‐expressed in certain cancer cells, implying that NK1L and NK1S may functionally affect each other [20]. NK1S has been suggested as a negative modulator of NK1L in a recent report, whereas there is no report defining signaling pathways affected by this modulation [8]. Our previous study has revealed that NK1R can form homodimers, implying that NK1L and NK1S may interact with each other to influence downstream signaling pathways and some disease states including cancer progression [21]. The gap in understanding functional properties of the receptors highlights an urgent need for further research to elucidate the interactive roles of these variants in signaling and cancer biology.

This study aimed to elucidate the molecular interaction between NK1L and NK1S using co‐immunoprecipitation and NanoBiT technology. The molecular crosstalk effects on downstream signaling and cellular responses were investigated using various biochemical and cell‐based assays. The findings of our study indicated the potential for these two isoforms to form a complex, with NK1S exhibiting a negative modulation of NK1L‐mediated G protein signaling through enhanced β‐arrestin recruitment. The crosstalk between NK1L and NK1S was further determined in SP‐stimulated migration of A549 cells endogenously expressing NK1R. The results provide a foundation for comprehending the functional interactions between NK1R variants.

## Materials and methods

### Materials

Substance P (cat. no. S6883) and cycloheximide (cat. no. C7698) were purchased from Sigma‐Aldrich (St. Louis, MO, USA) and prepared following the manufacturer's recommendations. Cell culture media (DMEM, RPMI‐1640, and Opti‐MEM) and fetal bovine serum (FBS) were acquired from Invitrogen (San Diego, CA, USA). All plasmids and reagents used for NanoBiT and HiBiT assays were provided by Promega (Madison, WI, USA). pcDNA3.1 vector was obtained from Invitrogen. SRE‐, CRE‐, and NF‐κB‐luciferase (luc) vectors were purchased from Stratagene (San Diego, CA, USA). Primary antibodies such as anti‐HA antibody (cat. no. ab9110) were obtained from Abcam (Cambridge, UK). Agarose beads conjugated with anti‐FLAG antibodies (cat. no. A2220) were bought from Sigma‐Aldrich (St. Louis, MO, USA). Anti‐ERK1/2 (cat. no. sc‐514302) antibodies and anti‐NK1R antibodies (cat. no. SC‐36591) were from Santa Cruz Biotechnology (Dallas, TX, USA). Anti‐pERK1/2 (Thr202/Tyr204) (cat. no. 4370) antibodies were from Cell Signaling Technology (Danvers, MA, USA). Secondary antibodies against the primary antibodies from rabbit or mouse were obtained from SeraCare (Milford, MA, USA). PNGase F and restriction enzymes were from New England Biolabs (Ipswich, MA, USA). All other reagents used in the experiment were from Sigma‐Aldrich unless otherwise stated.

### Cell culture

HEK293 (RRID: CVCL_0045) and A549 (RRID: CVCL_0023) cells were purchased from American Type Culture Collection (ATCC, Manassas, VA, USA), where identity had been verified by short tandem repeat (STR) profiling within the past 3 years. Cells were expanded from the original authenticated stocks and used at low passage numbers (<20 passages after receipt) to preserve cell line identity. All cell lines were routinely tested for mycoplasma contamination using a MycoAlert™ Mycoplasma Detection Kit (Lonza, Basel, Switzerland) and confirmed to be mycoplasma‐free prior to use in the experiments. HEK293 and A549 cells lacking NK1R were generated using a CRISPR/Cas9‐based gene knockout system as described previously [21]. HEK293 cells were maintained in Dulbecco's modified Eagle medium (DMEM), and A549 cells were cultured in RPMI‐1640. Both media were supplemented with 10% FBS, 100 U·mL^−1^ penicillin G, and 100 μg·mL^−1^ streptomycin and kept at 4 °C. These cells were maintained at 37 °C in a humidified incubator with 5% CO_2_.

### Plasmid construction

The promoter sequence of *Human cytomegalovirus* (CMV) in pcDNA3.1 was replaced with the promoter sequences of *Ubiquitin C* (UbiC) or *Herpes simplex virus thymidine kinase type 1* (HSV‐tk) to establish a relatively weak expression system. Transcription efficiency of each promoter was previously reported [22]. Constructs containing NanoBiT and HiBiT tags were effectively expressed by the UbiC promoter to optimize signal intensity in HEK293 cells. Additionally, HA‐ and FLAG‐tagged receptors were expressed under the control of the CMV promoter in the pcDNA3.1 vector. The NanoBiT construct of chimeric G protein (mini‐Gsq) established previously [23, 24] was utilized to monitor the interaction of Gαq protein and NK receptors. The gene constructs used in this study are presented schematically (Fig. S1).

### HiBiT assay

As described in our previous report [21], membrane expression of NK1R splicing variants was assessed using the Nano‐Glo® HiBiT extracellular detection system from Promega. All procedures were conducted according to by the manufacturer's instructions. Briefly, HEK293 cells were transiently transfected with varying amounts (0.5, 5, 10, 50, and 100 ng per well) of plasmids encoding N‐terminal HiBiT‐tagged receptors using 0.2 μL Lipofectamine 2000 (Invitrogen, CA, USA). The next day, the medium was replaced with 100 μL of serum‐free medium, and the plate was equilibrated at room temperature for 5 min. Then, 100 μL of Nano‐Glo® HiBiT extracellular detection solution containing 1 μL of LgBiT protein, 2 μL of Nano‐Glo HiBiT substrate, and 97 μL of Nano‐Glo HiBiT buffer was added to each well. The plate was incubated at room temperature for 4 min without shaking before performing measurements with a SpectraMax L plate reader (Molecular Devices, CA, USA).

### NanoBiT complementation assay for characterizing molecular interaction

Dimerization between NK1R splicing variants was assessed using the NanoBiT assay [21]. Briefly, HEK293 cells were seeded into 96‐well white plates at a density of 2 × 10^4^ cells·well^−1^. The following day, cells were transfected with 50 ng of NanoBiT‐tagged receptor plasmid and an additional 50 ng of either vector or NanoBiT‐tagged receptor plasmid using 0.2 μL of Lipofectamine 2000. At 24 h post‐transfection, the medium was replaced with 100 μL of Opti‐MEM and the plate was equilibrated at room temperature for 10 min before adding NanoBiT substrate. Baseline luminescence was recorded for 10 min using a SpectraMax L plate reader.

To assess interactions of NK1R splicing variants with either mini‐Gsq, β‐arrestin1, or FYVE, 50 ng of each NanoBiT‐tagged plasmid and 100 ng of either vector or untagged receptor plasmid were used for transfection with 0.4 μL Lipofectamine 2000. NanoBiT assay was performed as described above. At 10 min after recording baseline, 1 nm SP was added, and the luminescence signal was continuously measured for 1 h.

### Detection of intracellular calcium increases

Cytosolic Ca^2+^ changes were monitored using a NanoBiT‐based method as previously described in detail [25]. Briefly, HEK293 cells were transfected with a total of 120 ng of plasmids per well, comprising 30 ng each of C‐terminal SmBiT‐tagged calmodulin (CaM), the LgBiT‐tagged myosin light chain kinase 2 binding motif (M2S), untagged receptor, and an additional 30 ng of either vector or untagged receptor using 0.3 μL Lipofectamine 2000. Changes in luminescence signals were measured for 30 min after adding SP.

To assess receptor resensitization, transfected cells were pretreated with 50 μm of cycloheximide (CHX), a protein biosynthesis inhibitor, followed by incubation at 37 °C for 2 h. Cells were then stimulated with 100 nm SP for 10 min to induce receptor desensitization. Medium containing the ligand was gently washed out with phosphate‐buffered saline (PBS) and replaced with Opti‐MEM containing 50 μm CHX. Cells were placed in a 37 °C incubator to allow the receptor to be resensitized. After 1 h, the cell plate was equilibrated at room temperature for 10 min before adding the NanoBiT substrate. Baseline luminescence was recorded for 10 min. Afterwards, 1 nm SP was added, and luminescence was measured continuously for the following 30 min.

### Western blotting and co‐immunoprecipitation

All procedures were previously described [21]. Cellular proteins were extracted using lysis buffer (150 mm NaCl, 50 mm Tris–HCl pH 8.0, 1% Triton X‐100, 20 mm NaF) with a protease inhibitor cocktail (Roche, Indianapolis, IN, USA). After centrifugation at 21 200 ***g*** for 15 min at 4 °C, the protein amount of the supernatant was quantified using a Bradford protein assay kit (Bio‐rad, Hercules, CA, USA). Subsequently, 10 μg of proteins from each sample were separated by 10% SDS/PAGE and transferred to nitrocellulose membranes. These membranes were then blocked with 5% skim milk in Tris‐buffered saline containing 0.2% Tween 20 for 30 min and then incubated with specific primary antibodies (anti‐ERK1/2 and anti‐pERK1/2, 1:2000 dilution; anti‐HA, 1:10 000 dilution) overnight at 4 °C, followed by incubation with HRP‐labeled secondary antibodies (1:5000 dilution) for 1 h at room temperature. Protein bands were detected using an enhanced chemiluminescence kit (Thermo Fisher Scientific, Rockford, IL, USA). Relative band intensity of each blot was visualized and analyzed with E‐blot (Shanghai, China). To further analyze expression of the receptors, cell extracts were mixed with 2× sample buffer containing 8 m urea or treated with PNGase F to remove oligosaccharides from the receptor prior to SDS/PAGE.

Receptor dimerization was examined by co‐immunoprecipitation. Cells were seeded into 60‐mm dishes and transfected with 4 μg of plasmids which included HA‐tagged receptor or FLAG‐tagged receptor. At 36 h after transfection, cells were washed with cold PBS and lysed with 1 mL lysis buffer (150 mm NaCl, 50 mm Tris–HCl, pH 7.5, 10 mm KCl, 1% Triton X‐100, 10 mm NaF) for 30 min. After centrifugation at 21 200 ***g*** for 15 min at 4 °C, the supernatant was transferred to a new tube and incubated with agarose beads conjugated with anti‐FLAG antibodies at 4 °C for 2 h. After washing with lysis buffer, the precipitates were separated by 10% SDS/PAGE, transferred to a nitrocellulose membrane, and blotted with anti‐HA antibodies.

### Reporter gene assay

HEK293 cells were seeded into 24‐well plates at a density of 8 × 10^4^ cells per well. The following day, cells were transfected with a mixture of 400 ng of receptor plasmids and 50 ng of either SRE‐ or CRE‐ or NF‐κB‐Luc reporter gene using 1 μL of Lipofectamine 2000. At 24 h after transfection, cells were starved overnight in serum‐free DMEM. Subsequently, cells were treated with SP for 6 h before being lysed using 100 μL of lysis buffer. Luciferase activity of the cell extract was measured using a SpectraMax L plate reader.

### RT‐PCR (reverse transcription polymerase chain reaction)

RNA extraction, reverse transcription, and PCR amplification with optimized conditions were performed as described in previous reports [21]. Specific primers were as follows: NK1L (443 bp), F: AAGAGTGCTGCCCAGAAAAAGCCTTC and R: ACAGGCCGTAGTACCATTCGTTG; NK1S (132 bp), F: GGGCCACAAGACCATCTACA and R: AAGTTAGCTGCAGTCCCCAC; β‐actin (313 bp), F: CACTCTTCCAGCCTTCCTTC and R: CTCGTCATACTCCTGCTTGC. PCR products were separated by 1.5% agarose gel electrophoresis.

### Lentivirus generation and infection

NK1L, NK1S, and NK2 genes were inserted into FG12 lentiviral vectors, and HEK293T cells were transfected with the expression vector and accessory plasmids using the calcium phosphate transfection method. At 48 h after transfection, culture supernatants were harvested and stored at −80 °C. HEK293 or A549 cells were infected with the virus for stable expression of the gene with high transduction efficiency.

### Chemotaxis assay

Following the method previously described [21], A549 cells in 100 μL RPMI containing 0.1% FBS were seeded into the upper chamber of an 8‐μm pore size transwell plate (Corning Inc., Corning, NY, USA) at a density of 1 × 10^4^ cells per well. Next, 650 μL of RPMI containing 0.1% bovine serum albumin with or without 1 nm SP was added to the lower chamber. The plate was kept at 37 °C in a humidified CO_2_ incubator. After 24 h, non‐migrated cells in the upper chamber were removed using a wet cotton swab. Cells migrated through the filter were fixed with methanol, stained with hematoxylin and eosin, and then counted from four different areas of an optical microscopic field (100 × objective).

### Statistical analysis

All data are presented as the mean ± standard deviation (SD), which was calculated using GraphPad Prism 5 software (San Diego, CA, USA) [21]. Statistical significance was calculated using an unpaired *t*‐test for comparisons between two groups and a one‐way analysis of variance (ANOVA) with Bonferroni's test for comparisons between three or more groups. A *P*‐value of <0.05 (*P* < 0.05) was considered statistically significant. Unless otherwise indicated, all experiments were independently performed at least three times.

## Results and discussion

### Characterization of expression properties and their complex formation of NK1R variants

**Fig. 1:**
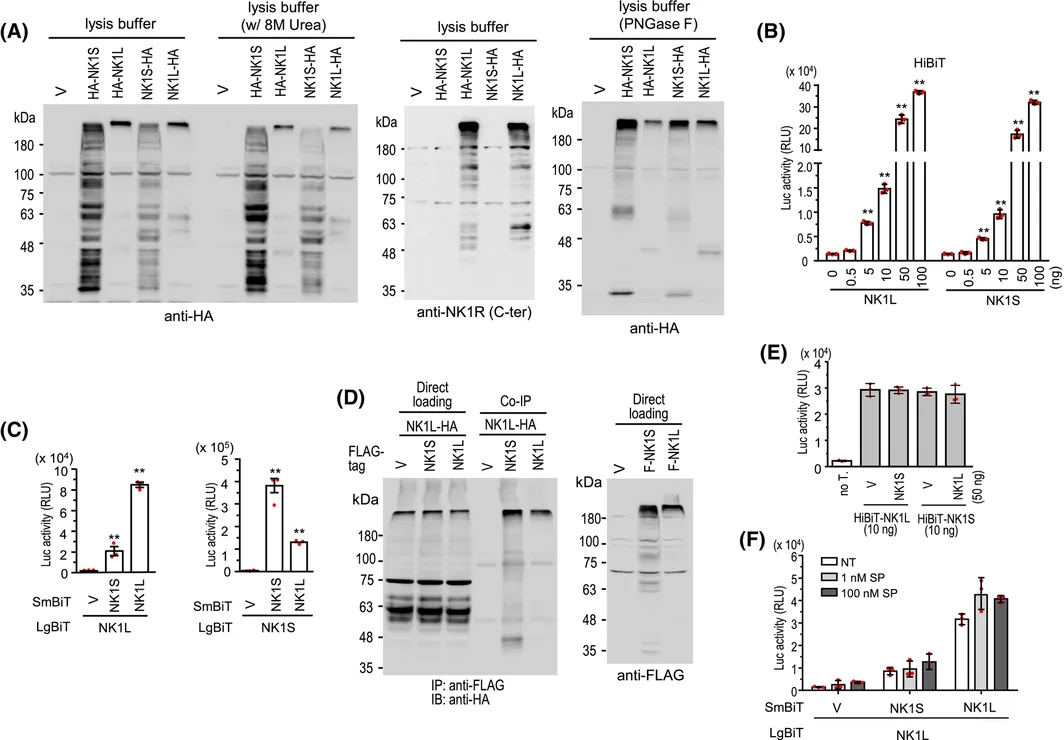
Expression patterns and molecular interaction of NK1R splicing variants, and their impact on G protein‐dependent signaling pathways. (A) Expression of HA‐tagged NK1R splicing variants. HEK293 cells were transfected with plasmid encoding HA‐tagged NK1L and NK1S at either the C or N termini. Cell extracts were subjected to western blot with anti‐HA antibodies. V, vector; NK1L, long form of NK1R; NK1S, short form of NK1R. (B) Cell surface expression of NK1R splicing variants. NK1L and NK1S were constructed with N‐terminal HiBiT tag in vectors driven by UbiC promoters. HEK293 cells were transfected with varying amounts of plasmid and used for the HiBiT assay. **P < 0.01 vs. vector (V). (C) Luminescence signal induced by receptor dimerization. Each variant was tagged with either LgBiT or SmBiT at the C terminus and co‐expressed in HEK293 cells. Changes in luminescence signal were recorded by NanoBiT assay. **P < 0.01 vs. vector (V). (D) HEK293 cells were co‐transfected with C‐terminal HA‐tagged NK1L along with N‐terminal FLAG‐tagged NK1L or NK1S. Cell lysates were incubated with anti‐FLAG agarose, followed by SDS/PAGE and western blotting with anti‐HA antibody. In direct loading, there is a nonspecific band at 75 kDa detected by anti‐HA antibody. (E) HEK293 cells were transfected with 10 ng HiBiT‐tagged receptor plasmids and 50 ng intact receptor plasmids and applied to HiBiT assay. (F) HEK293 cells transfected with the plasmids of SmBiT‐tagged receptor and LgBiT‐tagged receptor were treated with substance P for 30 min and applied to NanoBiT assay. All experiments were performed in triplicate and repeated at least 3 times. The data graphs are presented as mean ± SEM of the represented experiments. Statistical significance was calculated using an unpaired *t*‐test.

Splice variants of GPCRs have been observed to exhibit variation in terms of protein expression levels and membrane localization. Consequently, the initial phase of the study entailed the characterization of the expression properties of NK1R variants. To determine total protein levels, western blot analysis using anti‐HA antibodies was performed with HEK293 cells expressing the C‐ or N‐terminal HA‐tagged NK1Rs under the CMV promoter. As shown in Fig. 1A, both variants were effectively expressed regardless of the tagging position. However, their migration patterns on SDS/PAGE exhibited disparities. The presence of multiple diffuse bands is a common occurrence for a considerable number of GPCRs in SDS/PAGE, which can be attributed to the heterogeneous glycosylation of NK1R in HEK293 cells or the random refolding of the 7‐TM protein during the gel run (Fig. 1A left panel). When the cell extracts were blotted with antibody for the C‐terminal of NK1R, the migration pattern of NK1L was similar to that in the blot with anti‐HA antibody (Fig. 1A middle panel). Since this antibody was developed against the C‐terminal of the receptor, NK1S was not detected. To efficiently denature the receptors, 8 m urea was added to the cell extracts, but the migration pattern was not changed, implying that multi‐bands and overhanging above the running gel were not affected by the high concentration of urea. The cell extracts treated with PNGase F showed a much clearer pattern with overhanging bands, suggesting that the receptors are heavily glycosylated during migration to the plasma membrane (Fig. 1A right panel). NK1L exhibited a consistently higher molecular weight compared to NK1S, a phenomenon that was ascribed to the absence of the C‐terminal region in NK1S. The lowest bands of NK1L were located at around 45 kDa, and those of NK1S were below 35 kDa. As multi‐transmembrane proteins, NK1Rs were not easily extracted as a monomer with lysis buffer and stranded on the start line of the running gel; therefore, the intense bands were detected above 180 kDa (see all Western blots).

Given the documented tendency of GPCRs lacking a C‐terminal region to manifest defects in membrane trafficking [24, 26], we proceeded to undertake a comprehensive examination of the membrane expression levels of these two variants by performing a HiBiT assay in HEK293 cells, which were engineered to express receptors tagged with HiBiT at the N terminus. The expression of proteins was driven by the UbiC promoter, which exhibited a comparatively weaker strength compared to the CMV promoter. It is hypothesized that a weak promoter will result in a reduction of potential artifacts from the overexpression system. The level of HiBiT‐dependent luciferase activity exhibited a direct correlation with the quantity of plasmid present in HEK293 cells (Fig. 1B). Luminescence signals were comparable between cells expressing either NK1L or NK1S, indicating that both variants were effectively translocated to the cell membrane. Furthermore, it was determined that truncation of the C‐terminal region did not significantly affect membrane localization of NK1S.

Next, the complex formation between NK1L and NK1S was examined using the NanoBiT assay. Splicing variants tagged with SmBiT or LgBiT at the C terminus were co‐expressed in HEK293 cells. The results showed a marked increase in luciferase activity for NK1L/NK1L and NK1S/NK1S homodimers, while heterodimers NK1L/NK1S exhibited relatively lower activity, suggesting that receptors may exist as molecular complexes (Fig. 1C). Direct interaction between NK1L and NK1S was verified by co‐immunoprecipitation (co‐IP) analysis with cells expressing epitope‐tagged isoforms. The migration pattern of co‐precipitated NK1L‐HA in SDS/PAGE did not correlate with the band position of NK1L‐HA in the directly loaded sample, which may be due to conformational changes of the protein during the experiment. NK1L‐HA was precipitated with both FLAG‐NK1L and FLAG‐NK1S, which is mainly depicted as overhanging bands (Fig. 1D, left panel). NK1L/NK1S heterodimers exhibited stronger band intensity compared to NK1L/NK1L homodimers, suggesting that heterodimers may be more prevalent or more stable (Fig. 1D). The discrepancy between the results depicted in Fig. 1C,D is likely attributable to inherent methodological differences. NanoBiT has been demonstrated to facilitate the capture of dynamic and transient interactions within a native cellular context. In contrast, co‐IP has been shown to preferentially detect more stable complexes that withstand the effects of experimental lysis conditions. Taken together, these results suggest that heterodimerization between NK1L and NK1S may induce conformational changes that can stabilize the receptor complex. This, in turn, may potentially modulate downstream signaling pathways and/or alter receptor trafficking or recycling patterns compared to monomers.

To determine whether the splice variants mutually affect their membrane expression, HiBiT‐tagged constructs of each variant were expressed in HEK293 cells with or without co‐expression of the intact (untagged) form of the other variant. HiBiT‐dependent luminescence was comparable irrespective of co‐expression, indicating that NK1L and NK1S do not markedly alter each other's cell surface abundance (Fig. 1E). Furthermore, NanoBiT assays with C‐terminally tagged NK1L and NK1S showed similar levels of luciferase activity before and after SP stimulation, suggesting that homo‐ and heterodimer formation is constitutive and not detectably regulated by ligand (Fig. 1F).

### NK1S negatively modulates SP/NK1L‐mediated G protein‐dependent signaling

**Fig. 2:**
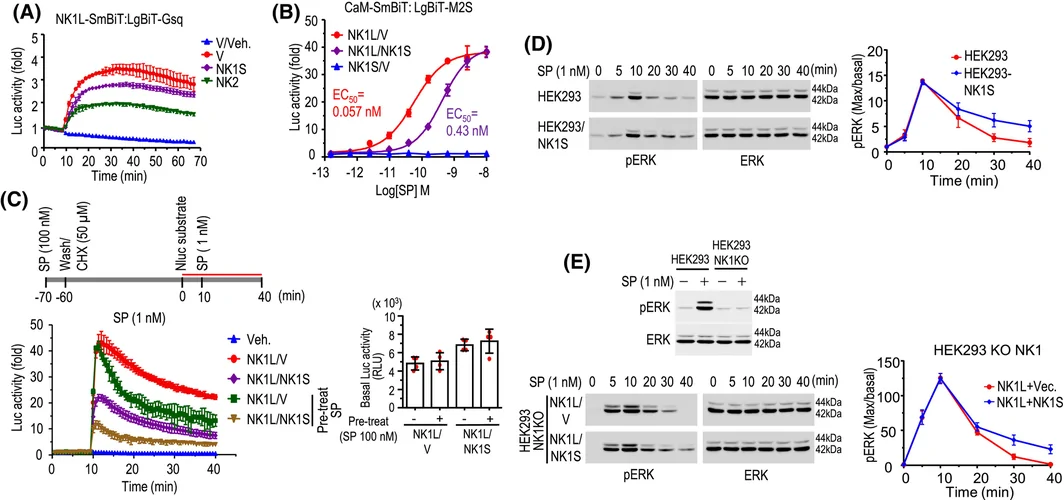
The effects of NK1S on NK1L‐mediated signaling. (A) Effect of NK1S and NK2 on SP‐induced NK1L and G protein interaction. HEK293 cells were co‐transfected with C‐terminal SmBiT‐tagged NK1L and N‐terminal LgBiT‐tagged mini‐Gsq, along with empty plasmid (V) or NK1S or NK2 receptor. All plasmids were expressed under the control of UbiC promoter. Cells were treated with vehicle (Veh) or 1 nm SP to stimulate NK1L‐G protein interaction, which was assessed by NanoBiT assay. (B) Dose‐dependent curves of SP‐stimulated cytosolic Ca^2+^ mobilization mediated by NK1L and/or NK1S. Cells expressing Ubic‐driven NK1R splicing variants along with NanoBiT‐based cytosolic Ca^2+^ probes. CaM, calmodulin; M2S, myosin light chain kinase 2; V, vector. (C) Effect of NK1S on resensitization of NK1L. Left graph: HEK293 cells were transfected with NanoBiT‐based cytosolic Ca^2+^ probes along with NK1L and either vector (V) or NK1S. Prior to experiments, cells were incubated with cycloheximide (CHX) for 2 h and maintained throughout the assay. To induce receptor desensitization, cells were pretreated with SP (100 nm) or Vehicle (Veh.) for 10 min, followed by thorough washing and 1 h incubation to allow receptor resensitization. Cells were then re‐stimulated with 1 nm SP. Luminescence was then monitored for 30 min using a luminometer. Right bar graph: Basal luciferase activity in groups pretreated with 100 nm SP (+) or vehicle (−). Luminescence was measured for 10 min right before re‐stimulation with 1 nm SP. (D, E) Time‐dependency of ERK phosphorylation response to SP. HEK293 wild‐type cells and cells exogenously expressing NK1S (D), or NK1R‐knockout HEK293 cells reconstituted with NK1L with or without NK1S (E) were treated with 1 nm SP. Cells were harvested at designated time points and subjected to western blotting with anti‐ERK or pERK antibodies. All experiments were performed in triplicate and repeated at least 3 times. The data graphs are presented as mean ± SEM of the represented experiments.

**Fig. 3:**
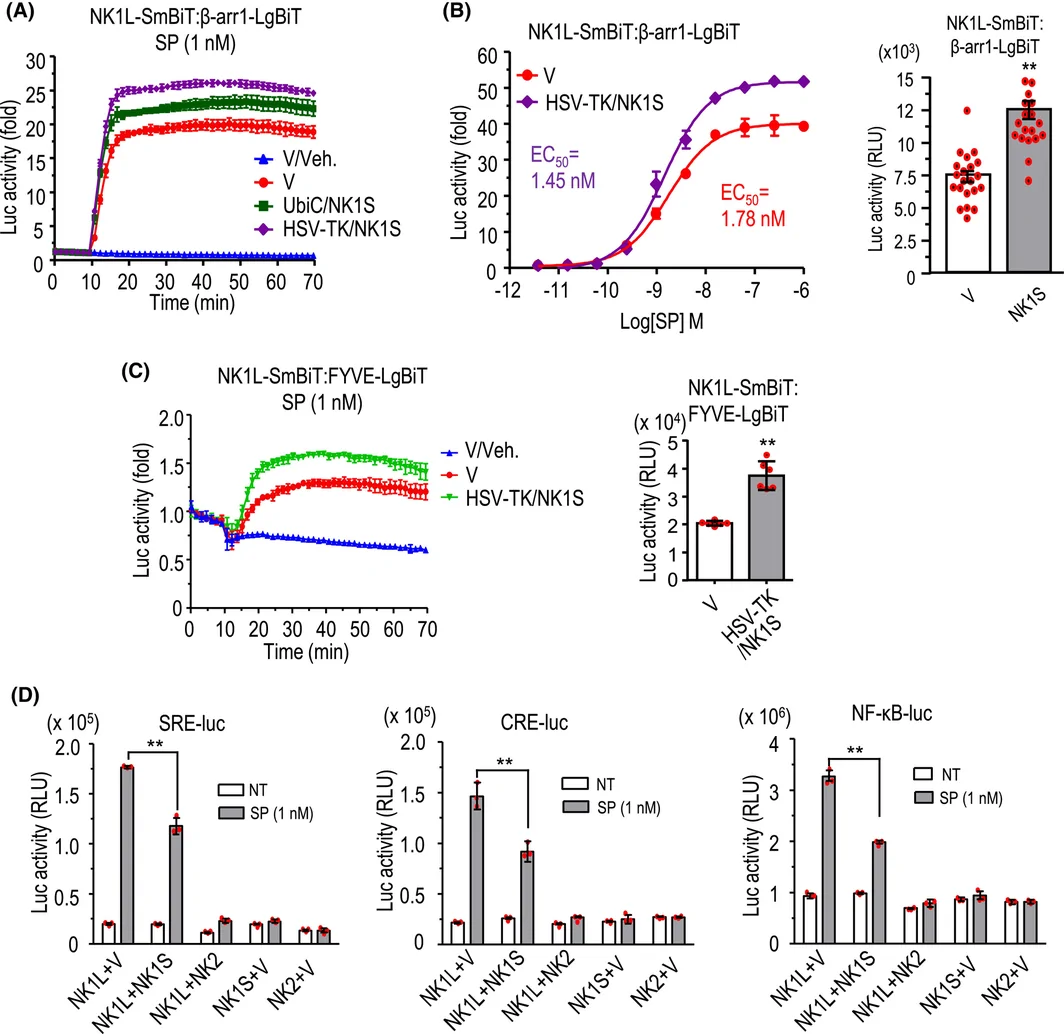
NK1S enhances β‐arrestin‐mediated NK1L internalization and suppresses SP‐induced cellular responses. (A) Luciferase activities of NK1L‐SmBiT and β‐arrestin1‐LgBiT in the presence of vector (V) or intact NK1S driven by different promoters (UbiC or HSV‐TK) following SP (1 nm) treatment. β‐arr1, β‐arrestin1; V, vector; Veh, vehicle. (B) SP‐induced β‐arrestin1 recruitment to NK1L in the presence of NK1S. HEK293 cells co‐expressing NK1L‐SmBiT and β‐arrestin1‐LgBiT, with or without HSV‐TK‐driven NK1S were treated with increasing concentrations of SP. Changes in luciferase activities were measured in real‐time. Left graph: Dose‐dependent curves of SP‐stimulated maximum luminescence signals; EC_50_, half maximal effective concentration. Right graph: Basal luciferase activities in cells expressing NanoBiT construct with or without intact NK1S before ligand stimulation. **, *P* < 0.01 vs. vector (V). (C) Effect of NK1S on SP‐stimulated NK1L internalization. Cells expressing NK1L‐SmBiT and FYVE‐LgBiT along with HSV‐TK‐driven NK1S were treated with SP (1 nm). Luciferase activities were measured for 60 min after treatment (left graph). Basal luminescence of each group was obtained before ligand stimulation. **P < 0.01 vs. vector (V). (D) NK1S partially inhibited SP‐stimulated SRE‐, CRE‐, NF‐κB‐luciferase reporter gene expression. HEK293 cells were transfected with different receptors alone or in combination, along with luciferase reporter plasmids. Cells were incubated with SP (1 nm) for 6 h before measuring luciferase activity with a luminometer. V, vector; Veh., vehicle; SRE‐luc, SRE‐luciferase; CRE‐luc, CRE‐luciferase; NF‐κB‐luc, NF‐κB luciferase. **P < 0.01 (NK1L + V vs. NK1L + NK1S). All experiments were performed in triplicate and repeated at least 3 times. The data graphs are presented as mean ± SEM of the represented experiments. Statistical significance was calculated using an unpaired *t*‐test.

To determine the effect of the receptor interaction, we examined the ability of NK1S to modulate the interaction between NK1L and an engineered mini‐Gsq protein, a chimera in which specificity‐determining residues in Gαs were replaced with those corresponding to Gαq [23]. The interaction between NK1L and mini‐Gsq was assessed using the NanoBiT assay. According to previous studies, the optimal combination for detecting the specified interaction is the fusing of SmBiT to the C‐terminal of the receptor and LgBiT to the N‐terminal of mini‐Gsq [21]. SP stimulation (1 nMm) induced robust luciferase activity in NK1L‐expressing HEK293 cells, indicating strong coupling with the Gαq protein (Fig. 2A). Co‐expression of NK1S resulted in a modest decrease in luciferase activity when compared to NK1L alone, although this effect was considerably less pronounced than the reduction observed with co‐expression of NK2, which was previously reported to negatively modulate NK1L signaling [21]. In order to further investigate the effect of NK1S on Gαq‐mediated signaling, SP‐induced cytosolic Ca^2+^ mobilization, a downstream event of the Gαq pathway, was examined using a NanoBiT‐based Ca^2+^ indicator [25]. In HEK293 cells that expressed NK1L, SP increased the cytosolic Ca^2+^ levels in a dose‐dependent manner, with an EC_50_ of 0.057 nm. This finding aligns with previous reports [21, 27] (Fig. 2B). Conversely, cells expressing NK1S alone exhibited no calcium response to SP, even at concentrations as high as 50 μm (data not shown), although it was previously reported that NK1S could induce calcium mobilization at micromolar concentration of substance P [8]. A notable observation was the rightward shift in the dose–response curve, with an approximately 7‐fold reduction in sensitivity to SP (EC_50_ = 0.43 nm), when NK1S was co‐expressed with NK1L. Taken together, these results demonstrate that NK1S can negatively affect the ability of NK1L to induce cytosolic Ca^2+^ mobilization, consistent with its interference with NK1L‐Gαq interactions. Nevertheless, given that NK1S has been shown to bind SP, these results do not exclude the possibility that NK1S‐mediated sequestration of SP reduces the availability of ligand for NK1L, thereby attenuating its response [8]. While ligand sequestration by NK1S may partially account for the reduced G protein signaling, this mechanism alone is unlikely to fully explain our observations. Consistent with this notion, isoform‐specific HiBiT assays showed that NK1S co‐expression does not measurably reduce NK1L surface abundance, arguing that the diminished Gαq/Ca^2+^ signaling is not mainly due to a loss of receptor expression at the plasma membrane. If NK1S functioned solely as a ligand sink, saturating concentrations of SP would be expected to rescue NK1L‐mediated Ca^2+^ responses; however, NK1S co‐expression continued to suppress signaling even at high SP concentrations (Fig. 2B). Moreover, the physical association between NK1S and NK1L (Fig. 1C,D) and the NK1S‐mediated enhancement of β‐arrestin1 recruitment to NK1L (Fig. 3A,B) strongly suggest that direct receptor–receptor interaction contributes to the modulatory effect through an allosteric mechanism, independent of ligand competition. Thus, both ligand sequestration and receptor–receptor modulation likely operate in concert, with their relative contributions depending on the NK1S‐to‐NK1L expression ratio and local SP concentration.

To examine the modulatory effect of NK1S on resensitization of NK1L, we next analyzed Ca^2+^ response after repeated SP stimulation (Fig. 2C, left graph). Cells that expressed NK1L alone or that were co‐transfected with NK1S were exposed to 100 nm SP or vehicle (control) for 10 min, followed by thorough washing. Subsequent to an interval of 1 h, during which receptor recycling to the plasma membrane occurred; the cells were once more challenged with 1 nm SP. In cells expressing NK1L alone, the maximal luciferase activity in response to 1 nm SP was comparable regardless of pretreatment with 100 nm SP or vehicle, suggesting that pretreatment with a high concentration of SP did not interfere with the ability of NK1L to resensitize and respond to subsequent stimulation. However, the sustained luciferase activity was significantly attenuated in SP‐pretreated cells compared to that in vehicle‐pretreated cells. This observation suggests that prior exposure to a high concentration of SP may induce partial desensitization, thereby reducing the duration of calcium response. In contrast, in HEK293 cells co‐expressing NK1L and NK1S, the SP‐induced luciferase activity was lower compared to cells expressing NK1L alone, consistent with the results shown in Fig. 2B. A notable finding was that this reduction was more pronounced in SP‐pretreated NK1L/NK1S co‐expressing cells compared to that of vehicle‐pretreated cells. This finding suggests that NK1S may interfere with the resensitization process, potentially by altering the balance between internalized and recycled NK1L and thereby limiting its availability at the plasma membrane for subsequent signaling. Basal luciferase activities remained constant between the vehicle‐treated and SP‐pretreated groups, indicating that residual ligand was absent and that intracellular Ca^2+^ levels were stabilized at this time, irrespective of ligand pretreatment (Fig. 2C, bar graph).

NK1R activation has been demonstrated to induce ERK phosphorylation, which is considered to be one of the most sensitive signaling responses to SP stimulation. In HEK293 cells, SP‐induced ERK phosphorylation reached its peak at 10 min after exposure and then gradually decreased to basal levels by 40 min via the endogenous receptor (Fig. 2D). In cells that expressed NK1S, the ERK phosphorylation exhibited a more sustained pattern, remaining elevated until 40 min after SP treatment (Fig. 2D; Fig. S2). This prolonged activation suggests that NK1S may modulate the signaling dynamics of ERK through mechanisms distinct from the transient response typically mediated by G protein‐dependent signaling. To further substantiate this observation, HEK293 cells deficient in NK1R were generated by CRISPR/Cas9. In the knockout cells, SP (1 nm) failed to induce ERK phosphorylation, confirming that SP‐induced ERK activation was NK1R‐dependent. The reintroduction of NK1L using lentivirus in NK1R knockout cells led to the restoration of SP‐induced ERK phosphorylation, exhibiting a pattern analogous to that observed in wild‐type cells, albeit with augmented intensity. This effect can be attributed to the comparatively elevated expression levels of NK1L (Fig. 2E, Fig. S3). Notably, the co‐expression of NK1S with NK1L in these knockout cells led to a sustained ERK phosphorylation response. This activation persisted through 40 min of incubation. It is noteworthy that the magnitude of ERK phosphorylation remained comparable between NK1L‐expressing and NK1L/NK1S co‐expressing cells. This finding suggests that NK1S may modulate the duration of ERK activation rather than its magnitude, possibly through the β‐arrestin‐mediated pathway. It is well established that β‐arrestins function as scaffold proteins for various signaling complexes, including the ERK pathway. They have been implicated in promoting sustained ERK activation independent of G protein signaling [28]. The sustained ERK activation observed in NK1L/NK1S co‐expressing cells suggests the possibility that NK1S may provide an opportunity for NK1L to recruit β‐arrestins, thereby prolonging ERK signaling, as previously described by Luttrell et al. [29].

### NK1S modulates SP‐induced NK1L trafficking in association with enhanced β‐arrestin recruitment

The subsequent investigation focused on elucidating the impact of NK1S on the interaction between NK1L and β‐arrestin following SP stimulation, employing the NanoBiT assay. HEK293 cells were co‐transfected with UbiC‐driven NK1L‐SmBiT, UbiC‐driven β‐arrestin1‐LgBiT, and different promoter‐driven intact NK1S. The cells were then treated with SP (Fig. 3A). SP elicited a heightened response in luciferase activity in the presence of intact NK1S relative to its absence. Notably, HSV‐TK‐driven NK1S exhibited greater luminescence than UbiC‐driven NK1S, despite HSV‐TK being a much weaker promoter. This suggests that NK1S‐mediated modulation of NK1L‐β‐arrestin1 interaction may be influenced by promoter‐dependent expression levels. Given that NK1S can bind to SP on the cell surface without transducing signals, higher expression of NK1S may interfere with the binding of SP and NK1L and the activation of NK1L by occupying SP. Consequently, reduced levels of NK1S expressed by HSV‐TK may modulate NK1L behavior by complexing with NK1L rather than by inhibiting the interaction of SP with NK1L. Dose‐dependent curves of β‐arrestin1 recruitment showed that NK1L recruited β‐arrestin1 more efficiently in the presence of HSV‐TK‐driven NK1S, as evidenced by an increase in maximal efficacy (Fig. 3B). Furthermore, the basal luciferase activity of NK1L‐SmBiT and β‐arrestin1‐LgBiT was augmented in NK1S co‐expressing cells. Despite the absence of the C‐terminal domain necessary for direct interaction with β‐arrestin, the co‐expression of NK1S and NK1L has been observed to increase the absolute number of SmBiT‐tagged forms, including NK1L‐SmBiT monomers, NK1L‐SmBiT/NK1L‐SmBiT homodimers, and NK1L‐SmBiT/NK1S heterodimers. These forms have been shown to recruit β‐arrestin1‐LgBiT in response to SP, thereby enhancing the luciferase activity.

The recruitment of β‐arrestin1 to receptors upon ligand stimulation precedes their internalization from the plasma membrane. Given that NK1S facilitated the interaction between NK1L and β‐arrestin1 while negatively modulating NK1L resensitization (Figs 2C, 3A), we investigated whether this effect was associated with altered receptor internalization and trafficking by assessing the interaction between NK1L‐SmBiT and FYVE‐LgBiT domain, an early endosomal marker. In accordance with prior observations, HEK293 cells co‐expressing NK1L and NK1S demonstrated a marginally augmented fold increase in luminescence after SP stimulation (Fig. 3C, left graph). A statistically significant increase in basal luciferase activity was observed in NK1L/NK1S‐co‐expressing cells in comparison to those expressing NK1L alone, even in the absence of SP (Fig. 3C, right bar graph). This finding indicates that NK1S may increase the association of NK1L with the FYVE domain even in the absence of ligand stimulation, suggesting a change in basal receptor–endosome interactions that could influence NK1L trafficking dynamics. Although we did not perform microscopic visualization of tagged NK1L in this study, the preserved cell surface expression and SP‐induced G protein signaling, together with the increased β‐arrestin1 and FYVE‐domain association, argue against a predominant role of gross mislocalization or irreversible recycling defects and instead support a more subtle modulation of NK1L trafficking dynamics by NK1S.

### NK1S suppresses SP‐induced NK1L‐mediated cellular responses

The activation of NK1R has been demonstrated to regulate gene expression through multiple mechanisms, including pathways that control SRE‐, CRE‐, and NF‐κB‐dependent transcription. To assess the effect of NK1S on NK1L‐mediated gene expression, reporter assays were conducted using plasmids harboring *SRE‐luc*, *CRE‐luc*, or *NF‐κB‐luc* genes (Fig. 3D). Treatment with 1 nm SP in HEK293 cells expressing NK1L alone induced robust luciferase activity by these promoters, demonstrating that NK1L is sufficient to activate the corresponding transcription factors. NK1S has been demonstrated to markedly curtail NK1L‐mediated transcriptional responses. This assertion is substantiated by the observation that the co‐expression of NK1S and NK1L led to a substantial diminution of the luminescence signal in all three reporters, as compared to NK1L alone. It is noteworthy that the negatively modulated effect of NK1S appeared to be less pronounced than that of NK2, which was previously reported to be a negative regulator of NK1L‐mediated G protein signaling [21].

**Fig. 4:**
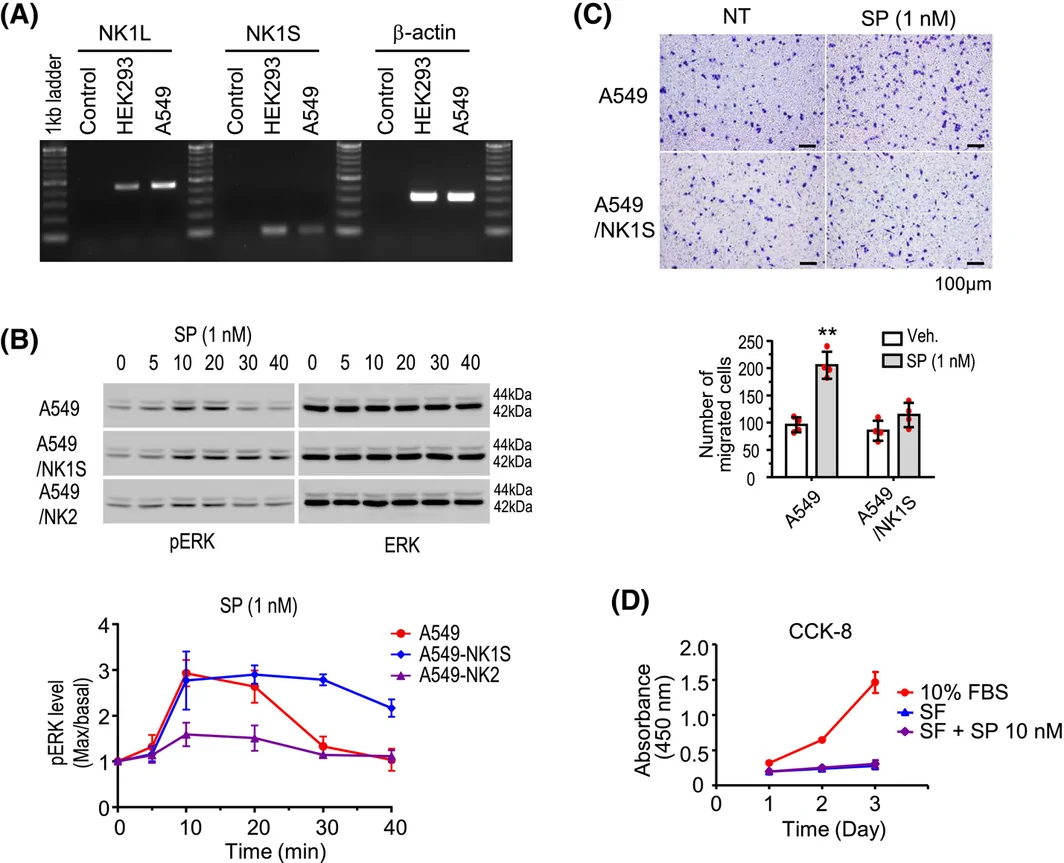
NK1S negatively regulates SP‐stimulated migration of A549 cells. (A) RT‐PCR for NK1R splicing variants with specific primers. Control indicates a blank PCR without reverse transcription products. (B) Effect of exogenous NK1S or NK2 on SP‐stimulated ERK phosphorylation in A549 cells. Wild‐type A549 cells or those exogenously expressing NK1S or NK2 were incubated with serum‐free media for 24 h and treated with 1 nm SP for varying time periods. After lysis, cell extracts were subjected to western blot with anti‐ERK or anti‐pERK antibodies (upper graph). The graph shows maximal ERK phosphorylation levels in comparison with basal levels, which were normalized with ERK blots at different time points (lower graph). (C) Migration of A549 cells in the presence of NK1S. A549 cells expressing exogenous NK1S were subjected to a chemotactic assay. Cells were loaded into upper wells of transwell migration chambers and 1 nm SP was added to lower wells. After 24 h of incubation, non‐migrated cells in the upper wells were removed with cotton swabs. Cells migrated to the lower surface of the transwell were fixed, stained, and counted under a microscope. **P < 0.01 (A549 vs A549/NK1S). (D) SP has no effect on proliferation of A549 cells. Cells were washed with serum‐free media and treated with 10% FBS or 10 nm SP. The measurement of cell growth was conducted by incubating the samples with CCK‐8 reagent for a period of 3 days. All experiments were performed in triplicate and repeated at least 3 times. The data graphs are presented as mean ± SEM of the represented experiments. Statistical significance was calculated using an unpaired *t*‐test.

A549 cells, a human lung carcinoma cell line, have been extensively utilized in previous research due to endogenous expression and functional activity of NK1R [16, 21, 30]. Given their suitability for studying the role of NK1R, we employed A549 cells to investigate the potential impact of the interaction between NK1L and NK1S on cancer cell behavior. RT‐PCR with variant‐specific primers indicated that both receptors might be expressed in A549 cells and that NK1L may be dominant (Fig. 4A). Due to suboptimal transfection efficiency observed in A549 cells, the introduction of lentivirus containing NK receptor genes was employed to achieve stable expression. SP induced transient ERK phosphorylation in A549 cells, thereby confirming the functional expression of endogenous NK1L. In accordance with the findings observed in HEK293 cells, the exogenous expression of NK1S in A549 cells resulted in prolonged ERK phosphorylation, persisting for up to 40 min (Fig. 4B). Conversely, exogenous expression of NK2 in A549 cells led to a substantial reduction in SP‐induced ERK phosphorylation, thereby validating our previous observation [21]. Furthermore, given the established role of SP in promoting cancer cell motility, we sought to examine how NK1S affected this process using a chemotactic assay. As shown in Fig. 4C, SP significantly stimulated A549 cell migration, confirming its role as a chemotactic factor for these cells. Unexpectedly, exogenous expression of NK1S led to a significant decrease in the number of migrated cells, suggesting that sustained ERK phosphorylation alone may not be sufficient to drive SP‐induced migration. In the context of cell growth, SP did not induce A549 cell growth in the absence of serum, indicating that SP exerts its influence on chemotactic migration rather than on cell proliferation (Fig. 4D).

The outcome of β‐arrestin‐mediated ERK activation is contingent upon the specific receptor and cell type. For instance, the migration of NIH3T3 cells induced by protease‐activated receptor‐2 (PAR2) required the β‐arrestin scaffolding of ERK at the plasma membrane, resulting in prolonged ERK activation and ensuing cytoskeletal remodeling [31]. In contrast, in COS‐7 cells expressing the angiotensin AT1α receptor, β‐arrestin retained ERK in the cytosol, preventing its nuclear translocation and limiting its ability to drive gene transcription, which may be necessary for migration [32]. It is noteworthy that both NK1S and NK2 negatively modulated NK1L‐mediated G protein‐dependent signaling, possibly by increasing β‐arrestin1 recruitment. However, the effects of these two proteins on ERK phosphorylation differed. Specifically, NK2 co‐expression led to decreased ERK phosphorylation, whereas NK1S co‐expression prolonged ERK activation. This finding indicates that NK1S and NK2 may regulate ERK through distinct mechanisms, potentially by influencing disparate β‐arrestin conformations, spatial localization, or by engaging alternative kinases.

The divergent outcomes of NK1S and NK2 on ERK phosphorylation and transcriptional activity suggest that NK1S promotes a biased signaling state at NK1L, favoring β‐arrestin–dependent over G protein–dependent ERK activation. This interpretation is supported by several converging lines of evidence in the present study: NK1S enhanced β‐arrestin1 recruitment to NK1L (Fig. 3A,B), sustained ERK phosphorylation without increasing its peak amplitude (Figs 2D, 4B, Fig. S4), and simultaneously reduced SRE‐, CRE‐, and NF‐κB‐driven transcriptional activity (Fig. 3D). Importantly, β‐arrestin–scaffolded ERK complexes have been shown to be retained in the cytoplasm, where they are unable to translocate to the nucleus and drive gene transcription [28]. This cytosolic sequestration of ERK provides a mechanistic basis for the apparent paradox observed in our study, that sustained ERK phosphorylation in NK1L/NK1S co‐expressing cells is paradoxically accompanied by reduced transcriptional reporter activity. Taken together, our findings are consistent with a model in which NK1S shifts NK1L signaling toward a β‐arrestin–biased, transcriptionally attenuated ERK pathway. The reduction in SP‐induced A549 cell migration observed upon NK1S expression further supports this interpretation, as nuclear ERK activity is likely required for transcriptional programs that drive cytoskeletal remodeling and chemotactic responses. Whether the sustained cytosolic ERK pool activated by NK1S confers alternative, non‐transcriptional functions such as regulation of cytoskeletal dynamics at the plasma membrane or modulation of endosomal signaling remains an important question for future investigation.

The physiological relevance of NK1S‐mediated modulation of NK1L is supported by the known co‐expression patterns of these isoforms in human tissues. NK1L and NK1S are co‐expressed in the brain as well as in peripheral organs including the lung and in various cancers. In the present study, HEK293 cells provided a heterologous system in which the NK1L‐NK1S stoichiometry could be experimentally controlled. Notably, lower NK1S expression more effectively enhanced β‐arrestin1 recruitment (Fig. 3A), suggesting that the relative abundance of the two isoforms is a key determinant of signaling output. In A549 lung carcinoma cells, which endogenously co‐express both isoforms with NK1L dominance (Fig. 4A), the NK1S‐mediated suppression of migration models a physiologically relevant scenario in which NK1S upregulation shifts the NK1L:NK1S balance toward attenuated G protein signaling. These observations support the view that NK1S is not merely a less efficient receptor, but a context‐dependent modulator whose functional impact varies according to tissue type, inflammatory status, and isoform stoichiometry [8, 10, 11, 12].

Taken together, this study provides a mechanistic framework for NK1R splice‐variant crosstalk, suggesting that NK1S functions as a context‐dependent biased modulator that attenuates G protein signaling while favoring β‐arrestin‐associated pathways through direct heterodimerization with NK1L. Elucidating how the NK1L:NK1S expression balance is regulated across tissues and disease states will be critical for understanding the full spectrum of SP signaling and for the rational design of NK1R‐targeted therapeutic strategies.

## Conflict of interest

The authors have no conflicts of interest relevant to this study.

## Author contributions

LPN, DTN, and JIH conceived and designed the project; LPN, DTN, MJ, JK, SI, TUN, and SH acquired the data; LPN, SH, BJP, and JIH analyzed and interpreted the data; LPN, DTN, JIH wrote the paper; BJP and JIH acquired funding.

## Supporting information


**Fig. S1.** Schematics of the protein constructs used in the experiments.
**Fig. S2.** Raw data for Fig. 2D.
**Fig. S3.** Raw data for Fig. 2E.
**Fig. S4.** Raw data for Fig. 4B.

## Data Availability

The data that support the findings of this study are available from the corresponding author [hjibio@korea.ac.kr] upon reasonable request.
