# Supplementary material for: The C‐terminal truncated splicing variant of NK1R negatively modulates substance P‐stimulated NK1R signaling
Source: FEBS Open Bio. 2026 Sep 21:10.1002/2211-5463.70334. Online ahead of print. doi: 10.1002/2211-5463.70334 (PMC13592011; doi:10.1002/2211-5463.70334)
Supplement: Supplementary file 1 [file FEB4-9999-0-s001.pdf]

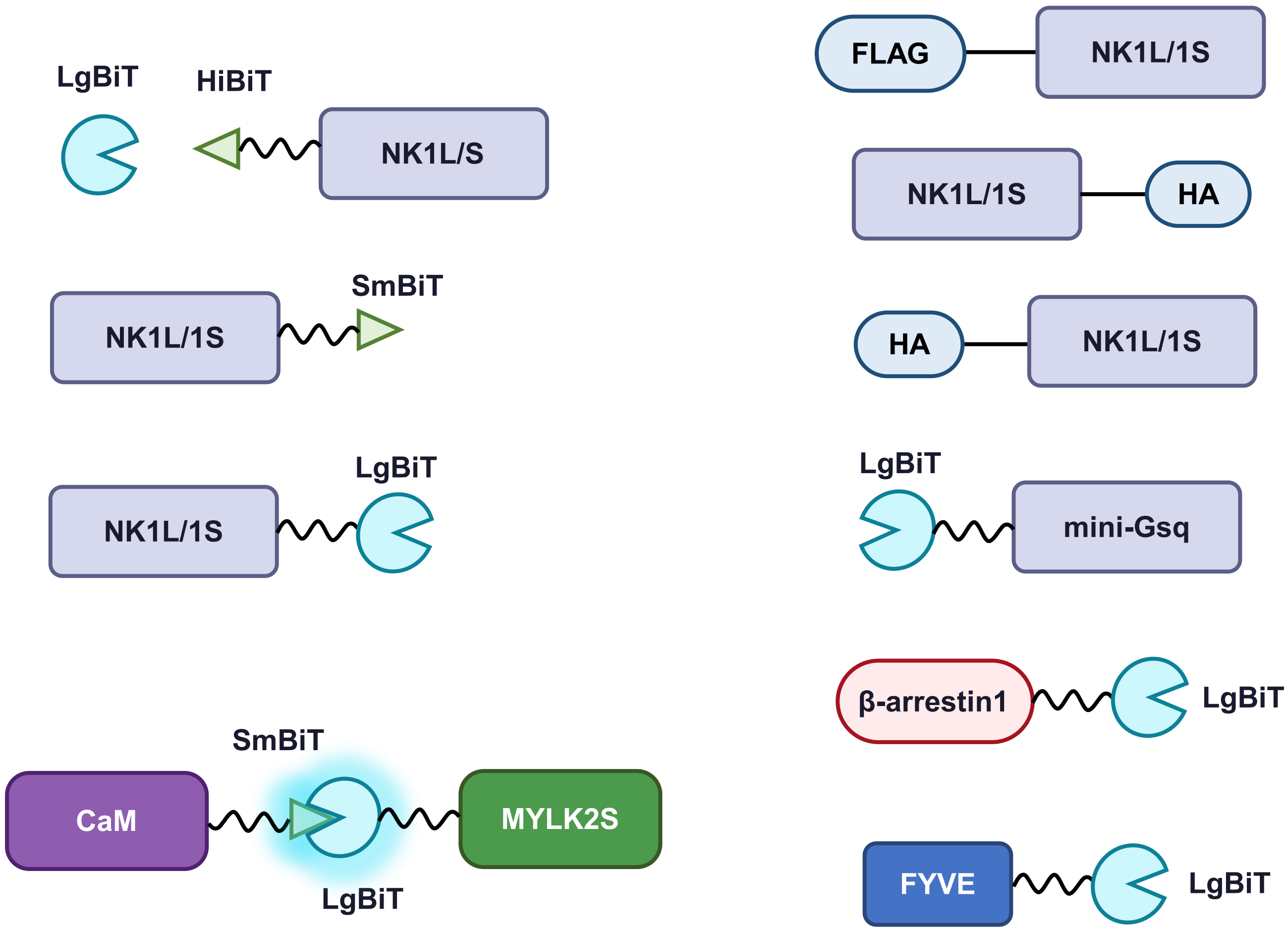

Supplementary Fig. 1 Schematics of the protein constructs used in the experiments

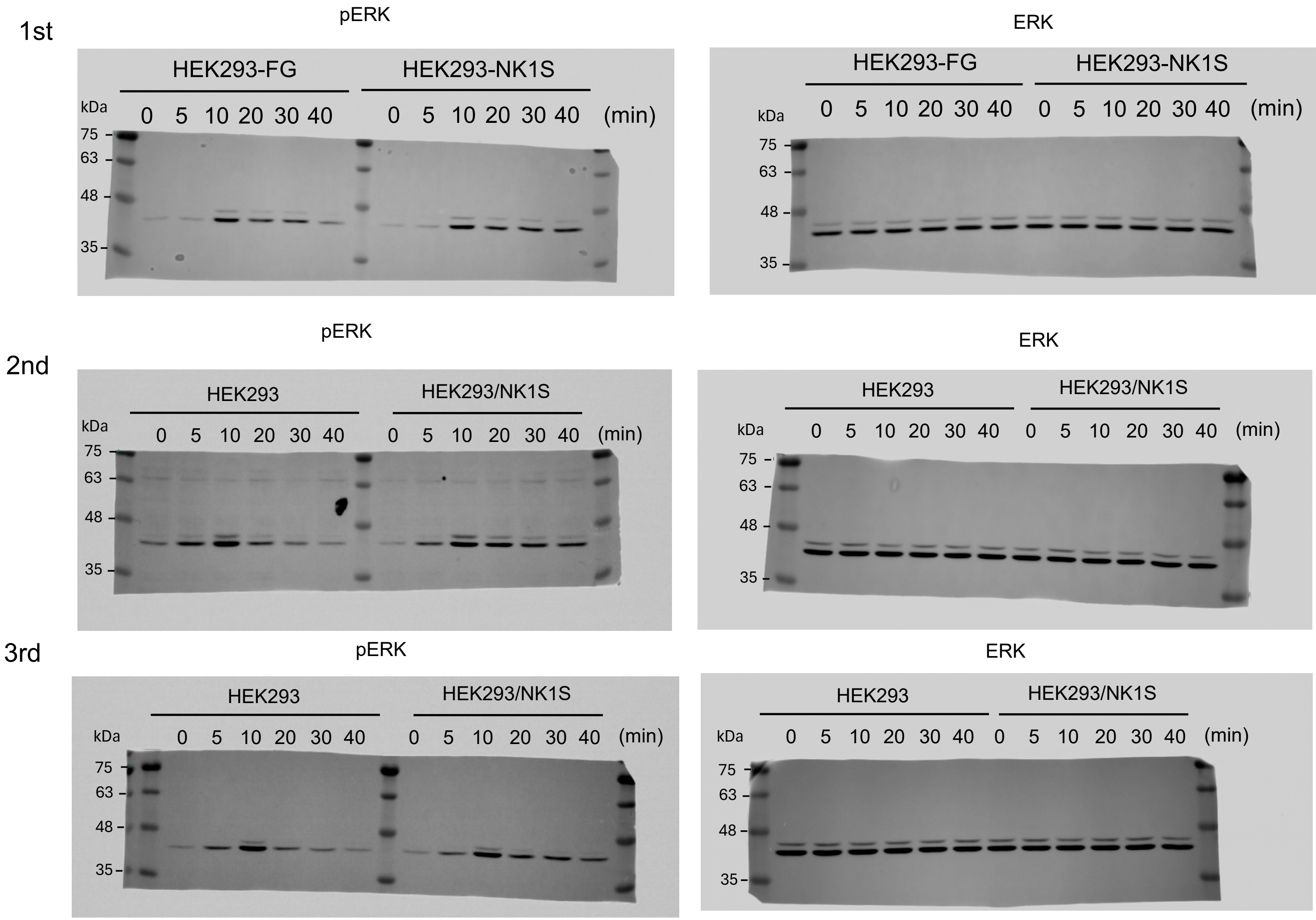

Supplementary Fig. 2 raw data for Fig. 2D

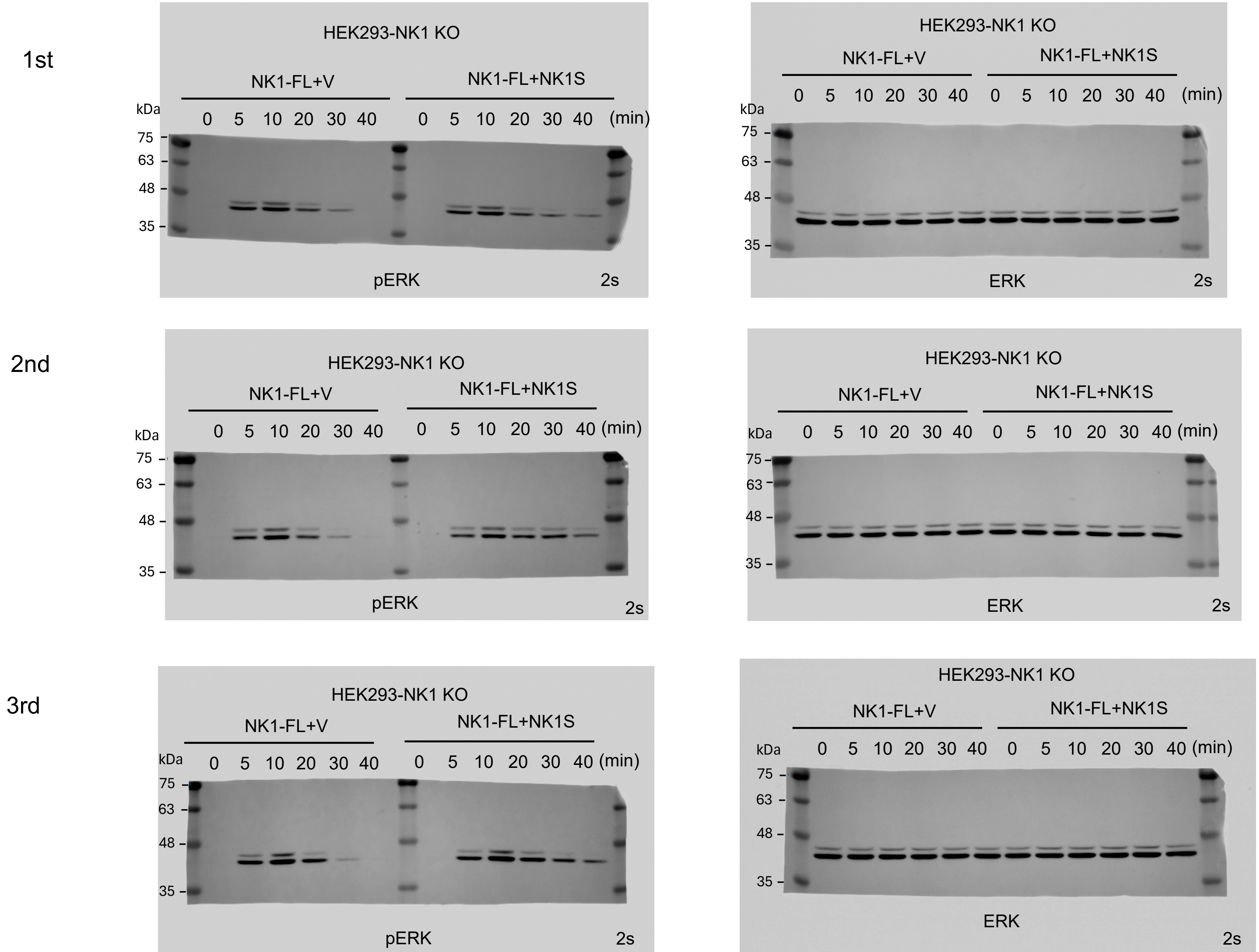

Supplementary Fig. 3 raw data for Fig. 2E

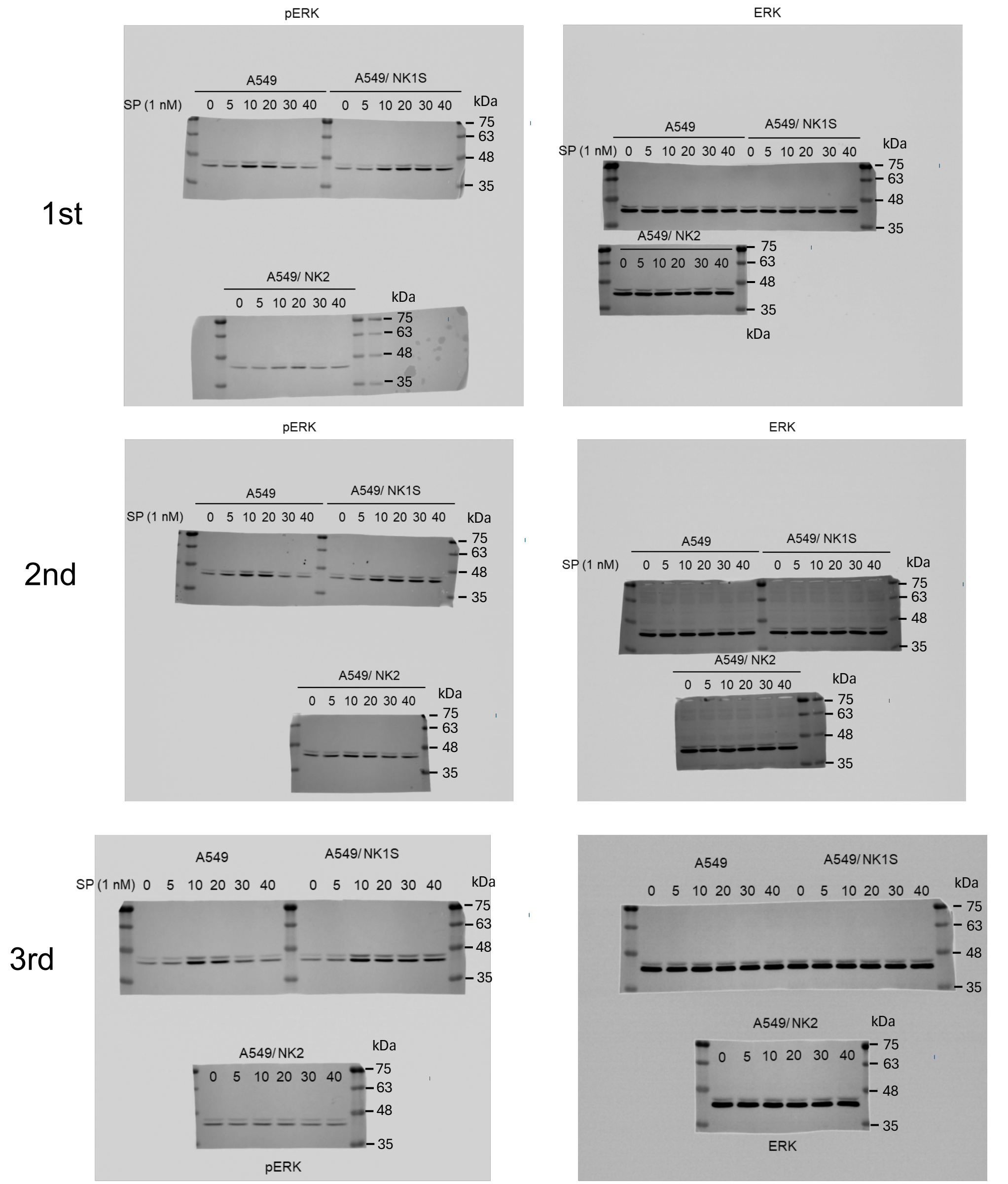

Supplementary Fig. 4 raw data for Fig. 4B
